# Considerations in performing hysterectomy secondary to uterus transplantation

**DOI:** 10.1111/aogs.14368

**Published:** 2022-04-20

**Authors:** Iori Kisu, Kouji Banno, Daisuke Aoki

**Affiliations:** ^1^ Department of Obstetrics and Gynecology Keio University School of Medicine Tokyo Japan

*Sir,*

We read with great interest the recent publication by Karlsson et al.^1^ regarding the surgical outcomes of hysterectomy after uterus transplantation (UTx), with a detailed description of graft failures. These novel perspectives based on the Swedish team’s experiences are very significant because hysterectomy is predetermined as an elective procedure due to the existence of several possible scenarios after UTx. Therefore, hysterectomy secondary to UTx should be performed with due consideration to the patient.

Hysterectomy may be (i) electively planned after live birth at cesarean section or later; or (ii) due to multiple failed pregnancy attempts, (iii) graft failures from insufficient blood flow or irreversible rejection, (iv) adverse events such as nephrotoxicity, infection or malignancies caused by immunosuppressants, or and (v) psychological burden. Moreover, a second pregnancy after raising a healthy child requires a thorough decision‐making process because of the limited recipient‐graft time.^2^ Since the timing of hysterectomy varies, exit strategies should be carefully defined to avoid exposure to unnecessary immunosuppressants or unexpected adverse events.

A major concern in UTx is the need for the removal of a failed uterine graft, as UTx is not a life‐supporting organ transplant and differs from conventional life‐supporting transplants in which graft removal generally results in a fatal outcome. In our experiments using cynomolgus macaques, the general conditions, including biochemical parameters, remained good in all animals when graft failures occurred because of ischemia and irreversible rejection.^3^, ^4^ In Karlsson et al.’s study,^1^ all three failed uterine grafts showed early ischemic signs, including reduced blood flow towards the inner uterine wall documented by Doppler studies. These cases eventually caused necrotic changes on uterine biopsy, leading to infectious episodes.^1^ In kidney transplantation, where hemodialysis can be introduced as an alternative life‐saving treatment, some controversial reports have opposed the removal of asymptomatic failed allografts due to the morbidity and mortality associated with post‐transplant nephrectomy.^5^ However, the uterine cavity is in contact with the external environment via the vagina. Hence, in a transplanted uterus, this may increase infection risks if blood flow insufficiency and rejection occur, resulting in septicemia and fatal outcomes unless hysterectomy is performed. The ensuing challenge is when to perform a hysterectomy to prevent such situations. Unlike other organs, the uterus is not life‐supporting. As there is no specific marker for uterine tissue, it is difficult to monitor graft rejection either clinically or with biochemical markers. Thus, it is important to evaluate early signs of ischemia with a cervical biopsy or Doppler assessments, to help in rapid decision‐making to perform hysterectomy, as the authors propose.

Another concern with hysterectomy secondary to UTx is that the uterus adheres to the surrounding tissues, leading to more time‐consuming and operative complications than in a regular hysterectomy. Furthermore, this operation may involve multiple laparotomies, and the burden (physical, psychological and financial) on the recipient is expected to be relatively high. Therefore, maximum consideration should be given to measures that avoid complications, such as placement of ureteric stents as well as multi‐faceted psychological care.

Although there is no consensus on the indications and decision‐making for hysterectomy after UTx, when performed following thorough consideration by each team, we hope that the criteria for these exit strategies will be established based on future clinical experience data.
